# Descriptor: *Synthetic Genomic Dataset With Diverse Ancestry (SynGen6)*

**DOI:** 10.1109/ieeedata.2024.3505852

**Published:** 2024-11-26

**Authors:** XINYUE WANG, SITAO MIN, JAIDEEP VAIDYA

**Affiliations:** 1 Center for Applied Statistics and School of Statistics, Renmin University of China, Beijing 100872, China; 2 MSIS, Rutgers University, Newark, NJ 07102 USA

**Keywords:** *All of Us*, diverse subpopulations, genomic analysis, synthetic genomic data

## Abstract

Advancements in genomic analysis techniques and data-driven research are driving precision medicine. However, in many cases, these advances are not equitable and do not help all subpopulations, since many existing genomic datasets lack diversity, limiting their applicability for studying populations beyond those of European ancestry. Thus, to advance genomic analysis and to allow for a fair benchmarking of novel proposed approaches, there is a significant demand for balanced and representative datasets. To address this issue, we developed, *SynGen6*, a synthetic dataset that encompasses six distinct populations, providing balanced representation across various ancestry groups. Using the *All of Us* dataset as a foundation, we utilized principal component analysis (PCA) and *ϵ*-local differential privacy (LDP) to generate synthetic samples while preserving genetic diversity and the privacy of individuals. To further enhance the dataset, we simulated phenotype vectors associated with significant single nucleotide polymorphisms (SNPs), mirroring real-world gene-disease associations. We also generated synthetic SNPs to watermark the dataset, enabling verification of cloud-based genomic computations for accuracy. Last, synthetic relatives were created to support research on kinship inference and family-based genomic analyses, resulting in a comprehensive dataset of 34 200 samples and 7120 SNPs across six populations. In this article, we describe the dataset and provide the Python scripts used to generate the dataset, which can be extended to create additional synthetic datasets, aiming to fuel advancements in genomic data analysis.

## BACKGROUND

Genomic data contain vast amounts of information about humans, which is essential for decoding the complex relationships between DNA and its environment, including disease mechanisms [1], protein functions [2], and metabolic pathways [3]. Traditionally, genomic data analysis involves a lengthy process of acquiring and preprocessing raw genetic data, using complex tools for both initial and subsequent analyses, and interpreting the results. Although recent advancements in genetic sequencing technologies and machine learning models have improved the efficiency of these processes, significant challenges remain. One major obstacle is the availability of high-quality datasets. On the one hand, genomic data are highly sensitive, and its use and sharing across institutional boundaries are tightly regulated due to privacy concerns. On the other hand, existing genomic datasets lack diversity and are not fully representative of the global population [4]. This lack of diversity is largely due to historical biases in data collection, with the majority of samples coming from individuals of European ancestry [4]. Furthermore, the complexity of genomic datasets—both in terms of their scale and varied formats—poses additional challenges in making this data widely accessible and usable. For example, massive sequencing datasets stored in repositories such as the Sequence Read Archive (SRA) [5] are difficult for many researchers to utilize effectively. Accessing and reanalyzing archived data typically requires extensive storage capacity, time-consuming downloads, and compute-intensive processing [6]. Consequently, valuable data often go unused [7], further limiting the advancement of genomic research. Given these challenges, a simpler, synthetic dataset with controllable features, such as customizable population distributions, could be highly valuable for preliminary research. Synthetic datasets can provide a more accessible and manageable alternative, enabling researchers to explore and test genomic analysis methods without the need for vast computational resources.

While deep generative models hold promise for capturing high-dimensional and complex distributions, their effectiveness in generating genomic data remains unclear [8]. Moreover, their “black-box” nature raises concerns, particularly in high-risk clinical applications where interpretability is crucial [9]. In this work, we adopt a simpler, feature-based approach. Specifically, we strategically create a synthetic genomic dataset that is well suited for developing and testing genomic data analysis methods. This dataset includes simplified and well-defined population structures and genetic information, which reduces privacy concerns and facilitates faster data sharing. This approach leverages the highly comprehensive *All of Us* dataset [10], though it could be applied to any similar alternative real dataset. *All of Us* contains nearly 250 000 whole genome sequences, along with integrated data from surveys, physical measurements, electronic health records (EHRs), and wearable devices. The dataset generation process follows a structured approach: we begin by performing standard quality control steps on the original *All of Us* dataset. Next, a meticulous procedure is applied to generate synthetic samples that replicate the characteristics of real genomic data while allowing for precise control over population representation. Specifically, we use a variant of LDP to perturb the original SNP values, generating new SNP sequences while preserving ancestry information by conditioning the perturbation on known ancestry for each sample. Overall, this synthetic dataset is carefully designed to maintain both the diversity and complexity found in real-world populations, allowing it to effectively simulate real-world genomic scenarios and to test a wide range of genomic analysis methods in a controlled environment. To build a dataset ideal for preliminary genomic data analysis, we outline the following key desiderata.

1. *Diversity of Populations*: The dataset should encompass a broad spectrum of populations to reflect real-world diversity accurately.
2. *Preservation of Genetic Information*: The dataset should retain the rich genetic information present in the original dataset.
3. *Complexity of Attributes and Values*: The dataset should demonstrate variations in both attributes and values. Each sample should include fundamental SNP data as well as more specific attributes such as phenol-type information. Moreover, the dataset should capture relationships among samples to support a wide range of genomic research.
4. *Exclusion of Errors*: The dataset should be of high quality, excluding any misleading information, particularly in the context of high-risk clinical research.
5. *Minimization of Privacy Risks*: While the privacy of samples in the *All of Us* dataset is protected using access control, the creation and use of the synthetic data should ensure that privacy risks are not increased for the participants.

## COLLECTION AND DESIGN

**FIG. 1.**
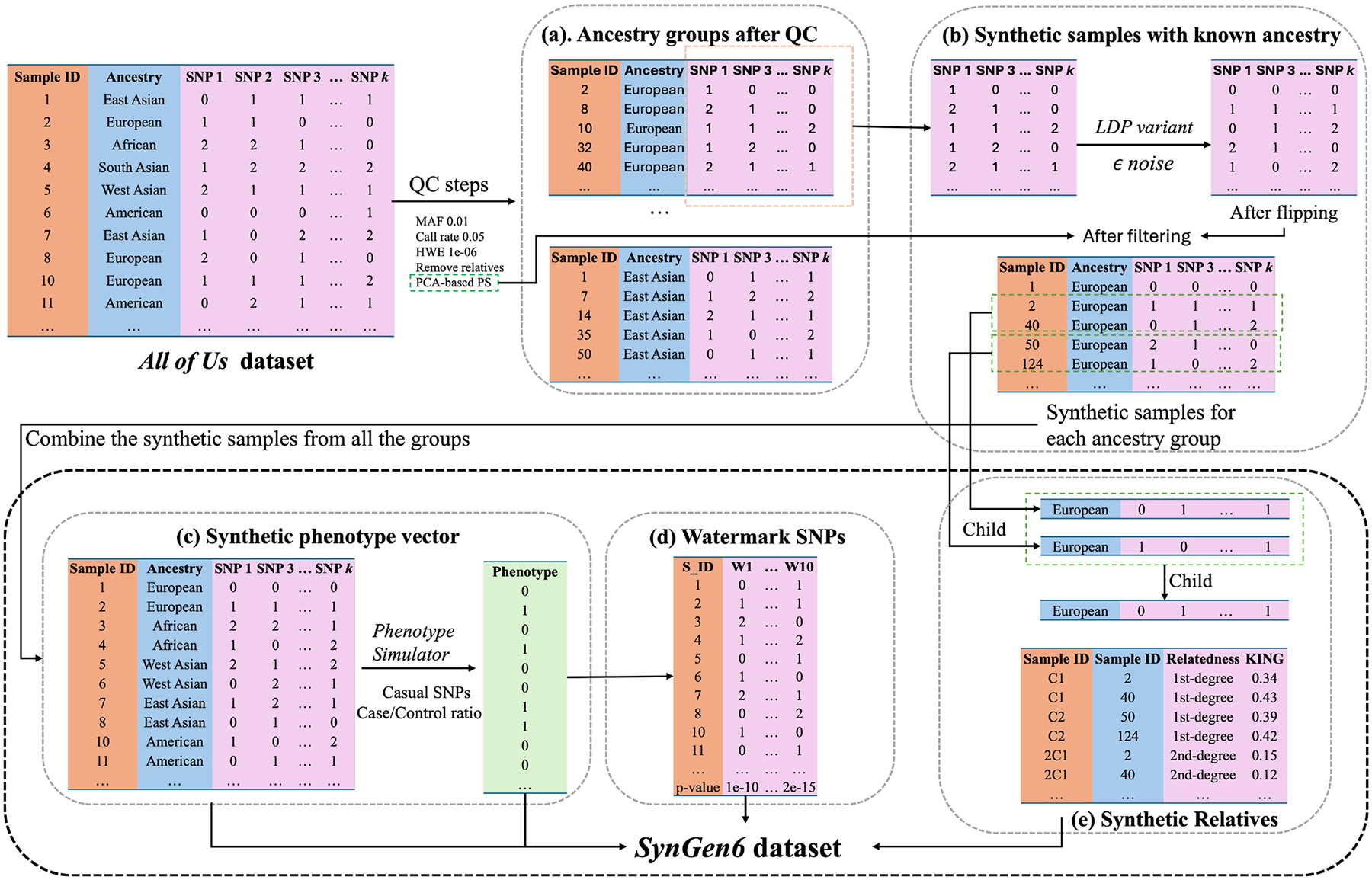
*SynGen6* generation methodology overview. (a) Standard QC steps on *All of Us* dataset. (b) Creation of synthetic samples using PCA and a variant of *ϵ*-LDP for each population. (c) Simulation of phenotype vector with predefined selected causal SNPs and case/control ratio. (d) Creation of watermark SNPs using the phenotype vector. (e) Creation of synthetic relatives following Mendel’s law.

The general methodology for generating the synthetic dataset is depicted in Fig. 1. The process begins with a quality control step applied to the original *All of Us* dataset to ensure data integrity. Following this, synthetic samples are generated for each subpopulation after conducting a PCA-based population stratification to control for population structure. Careful control over the number of samples ensures proportional representation without over-representing any particular population. Next, a phenotype vector is simulated using *PhenotypeSimulator* [11] on the combined synthetic and original datasets. The level of phenotype heterogeneity is controlled by a hyperparameter, which is set to 0.5 in this dataset, representing a balanced case/control ratio. Synthetic SNPs are also introduced to watermark the dataset, which can be used to confirm the integrity of the analysis results in the cloud settings [12]. Additionally, a random selection of samples from each subpopulation is chosen, and synthetic relatives are generated to simulate realistic familial structures.

## Preprocessing on *All of Us* Dataset

Initially, the *All of Us* dataset contains 245 394 samples and 202 959 SNPs. We begin by performing standard quality control steps [13] using PLINK 1.9 [14], which include filtering out SNPs with minor allele frequency (MAF) values smaller than 0.01, SNPs with a missing rate greater than 5%, and deviations from Hardy–Weinberg equilibrium (with the threshold chosen to be 1e − 6). Additionally, samples with a missing rate greater than 5% are removed to ensure data quality. We then perform PCA on the filtered genome dataset to extract the eigenvalues and eigenvectors, focusing on the top-200 principal components. Based on the ancestry information for each sample, we partition the dataset into six subsets, each representing a distinct subpopulation. After completing these steps, the final dataset contains 184 712 samples and 7180 SNPs and represents six different subpopulations, including 51.3% of European, 25.5% of African, 18.6% of American, 2.7% of East Asian, 1.4% of South Asian, and 0.4% of West Asian, ready for further analysis.

### Samples Generation With Known Ancestry

As mentioned in the previous section, one critical issue with existing genomic datasets is the lack of full representation across diverse populations. To address this and create a more balanced dataset, we aim to generate synthetic samples with known racial or ancestry information. For each subset of the dataset, we randomly select a group of real samples and perturb their records to produce synthetic samples. While there are several approaches to perturbation, we selected a variant of local differential privacy (LDP) [15] for two key reasons. First, differential privacy (DP) is a well-established privacy framework that provides formal guarantees ensuring that the distribution of query results changes minimally with the addition or removal of a single record in the database. LDP [16] further extends this notion by enabling privacy preservation at the individual level, particularly in scenarios where data are shared. Second, this variant of LDP allows for better preservation of the “closeness” between the original and synthetic samples, a key requirement when generating race-controlled synthetic genomic data at the expense of offering weaker privacy guarantees than standard LDP. Standard *ϵ*-LDP can be achieved by flipping the values of the SNPs based on a probability *q* = (1*/e*^*ϵ*^ + *d −* 1), where *d* represents the number of possible states for a SNP, and *ϵ* determines the noise level. For SNPs, we use an additive encoding, where the values 0, 1, and 2 represent homozygous dominant, heterozygous, and homozygous recessive, respectively, hence d = 3. There is a probability *q* that a state “0” SNP can be flipped to “2” and vice versa, which would significantly alter the genetic information and compromise the integrity of the synthetic data.

In the variant we adopt, to preserve more realistic genetic variation, we modify the flipping process as follows.

1. If $x_{k}^{j}=0$ or $x_{k}^{j}=2$, the state of SNP *k* is flipped to 1 with probability *q* = (2*/e*^*ϵ*^ + *d −* 1).
2. If $x_{k}^{j}=1$, the state of SNP *k* is flipped to 0 or 2 with probability *q* = (1*/e*^*ϵ*^ + *d −* 1).

This approach ensures that direct transitions between states “0” and “2” are avoided. By carefully selecting appropriate noise level, the synthetic samples retain the ancestry characteristics of the original dataset. To further ensure the privacy and quality of the generated data, we filter out low-quality synthetic samples—those that are either identical to the original ones or significantly deviate from the target ancestry group.

In our implementation, we set *ϵ* to 6 for each group—note that since every sample can only belong to one group, the overall *ϵ* is also 6 (due to parallel composition). To ensure balance in the final dataset, we manually control the number of synthetic samples generated for each group, targeting 5000 samples per group. This process results in a dataset containing six racial/ancestry groups with a total of 30 000 samples. If any subset of the original data contains fewer than 5000 samples, we repeat the process multiple times to generate a sufficient number of synthetic samples. Once the genotype arrays are generated, we create a list of random numbers to serve as the sample IDs. The samples are then sorted in ascending order based on their corresponding sample IDs.

### Phenotype Generation

After completing the previous steps, we proceed to simulate the phenotype vector for both real and synthetic samples. The primary objective is to assess the efficiency and effectiveness of techniques for gene-disease association, such as genome-wide association studies (GWASs). To create the synthetic phenotype vector, we utilize *PhenotypeSimulator* [11], a R/CRAN package for simulating multitrait, multilocus genotype-to-phenotype relationships. This tool enables the simulation of genetic variant effects, infinitesimal genetic effects (genetic background), nongenetic covariate effects, and noise effects with a predefined covariance structure. It also allows specification of the variance contribution of each component to the total phenotypic variance. In this work, we chose a combination of genetic effects and observational noise effects, which is standard for genetic association studies. Details of the simulation procedure are publicly available.^1^ In this tool, two key factors drive phenotype generation: the selection of significant SNPs and the number of cases (i.e., positive labels). To mimic a real-world scenario where a subset of the genotypes are likely correlated with the phenotype, we randomly select 10% (i.e., 718) SNPs and then utilize a logit model that uses these with randomly picked coefficients to generate a phenotype vector. This is likely to generate a phenotype that is correlated with these SNPs. We also adopt a balanced case/control strategy, ensuring equal representation of cases and controls.

### Watermark SNPs Generation

With the growing adoption of cloud computing for conducting GWAS, it becomes crucial to verify that a third-party server performs the computations outsourced to it correctly. Following the approach in [12], we propose generating several synthetic SNPs that are highly correlated with the simulated phenotype vector to “watermark” the dataset. This allows easy detection of any incorrect or deteriorated behavior from the server by comparing the returned results with the known results on the synthetic SNPs, at least for GWAS. In this work, we adopt the first approach from [12] due to its simplicity and efficiency. This method involves four key steps to generate synthetic SNPs. First, the synthetic SNP is initialized by duplicating the phenotype vector. Second, we identify two sets of indices: 1) where the phenotype is 1; and 2) where the phenotype is 0. Third, we determine the number of 0, 1, and 2 alleles required in the synthetic SNP. Finally, the initialized SNP is randomized based on the numbers calculated in the third step. After generating the synthetic SNPs, we assign random SNP IDs to each. In the implementation, we followed the hyperparameter settings from [12], with eps = 0.3 and *z*_0_ = 0.49, and generated 20 additional watermark SNPs, considering a balance between detection capability and overhead.

### Synthetic Relatives Generation

Several important tasks in genomic data analysis, such as kinship inference methods and family-based GWAS, require family relationship information. However, in real-world datasets, this information is often difficult to collect. For example, the original *All of Us* contains only around 6% of samples to be related by kinship. To overcome this limitation, our goal is to generate synthetic relatives to supplement the dataset. In particular, for each subset (i.e., samples with the same ancestry information), we randomly select 400 samples that do not have known relatives in the original dataset and generate synthetic relatives following Mendel’s law of inheritance. Given two SNP sequences from unrelated individuals, we simulate their offspring’s SNP sequence according to Mendel’s law. For example, if SNP 1 values for the two individuals are 0 and 1, the offspring’s SNP 1 value is either 0 or 1, with equal probability. In the synthetic genome array (produced after “Samples Generation With Known Ancestry” section), we randomly select 200 samples to represent fathers and 200 samples to represent mothers from each population. Using Mendel’s law, we simulate the first-degree descendants for these 200 parent pairs. Next, the first-degree descendants are randomly reassigned into father and mother groups, and the same process is repeated to generate 100 second-degree descendants. Finally, we repeat this process to produce 50 third-degree descendants. As a result, the dataset includes 200 first-degree, 100 second-degree, and 50 third-degree relatives per group. This process results in a dataset containing 7200 pairs (approximately 20% of the entire dataset) of related samples.

## VALIDATION AND QUALITY

**FIG. 2.**
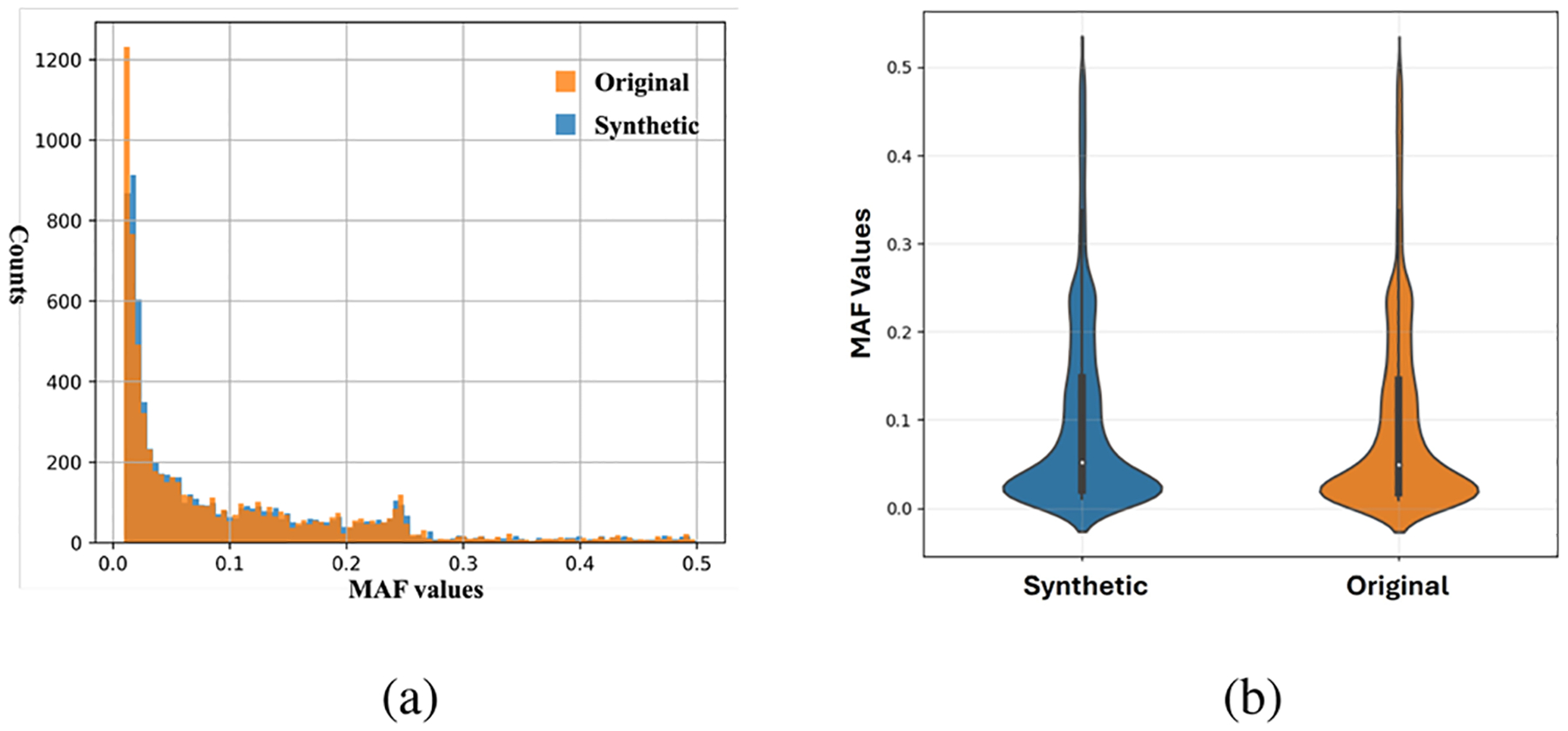
MAF values of original (orange) and synthetic (blue) SNPs. (a) Histogram. (b) Violin plot.

**FIG. 3.**
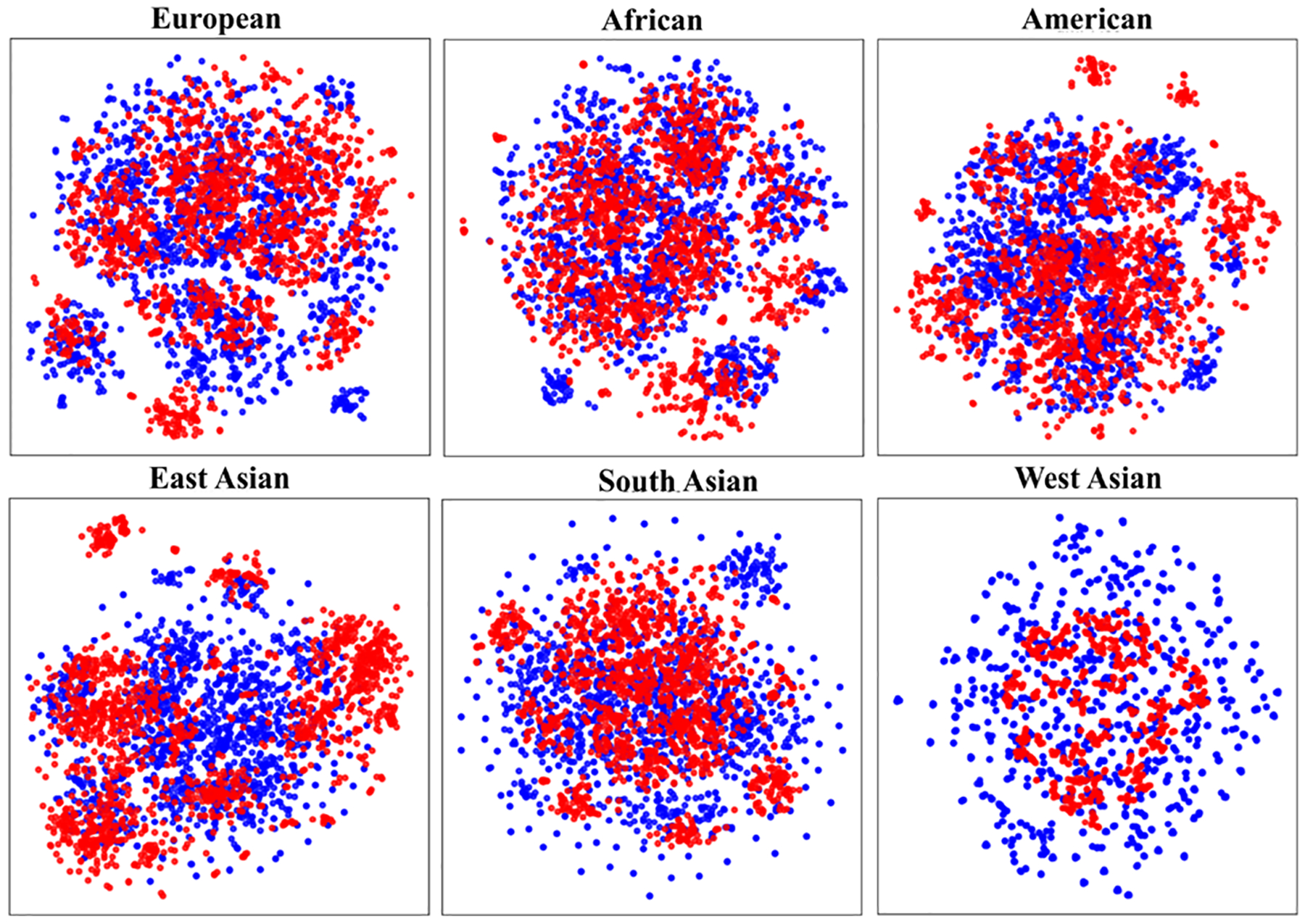
t-SNE plots across six groups of samples. Red dots indicate original samples. Blue dots indicate synthetic samples.

**FIG. 4.**
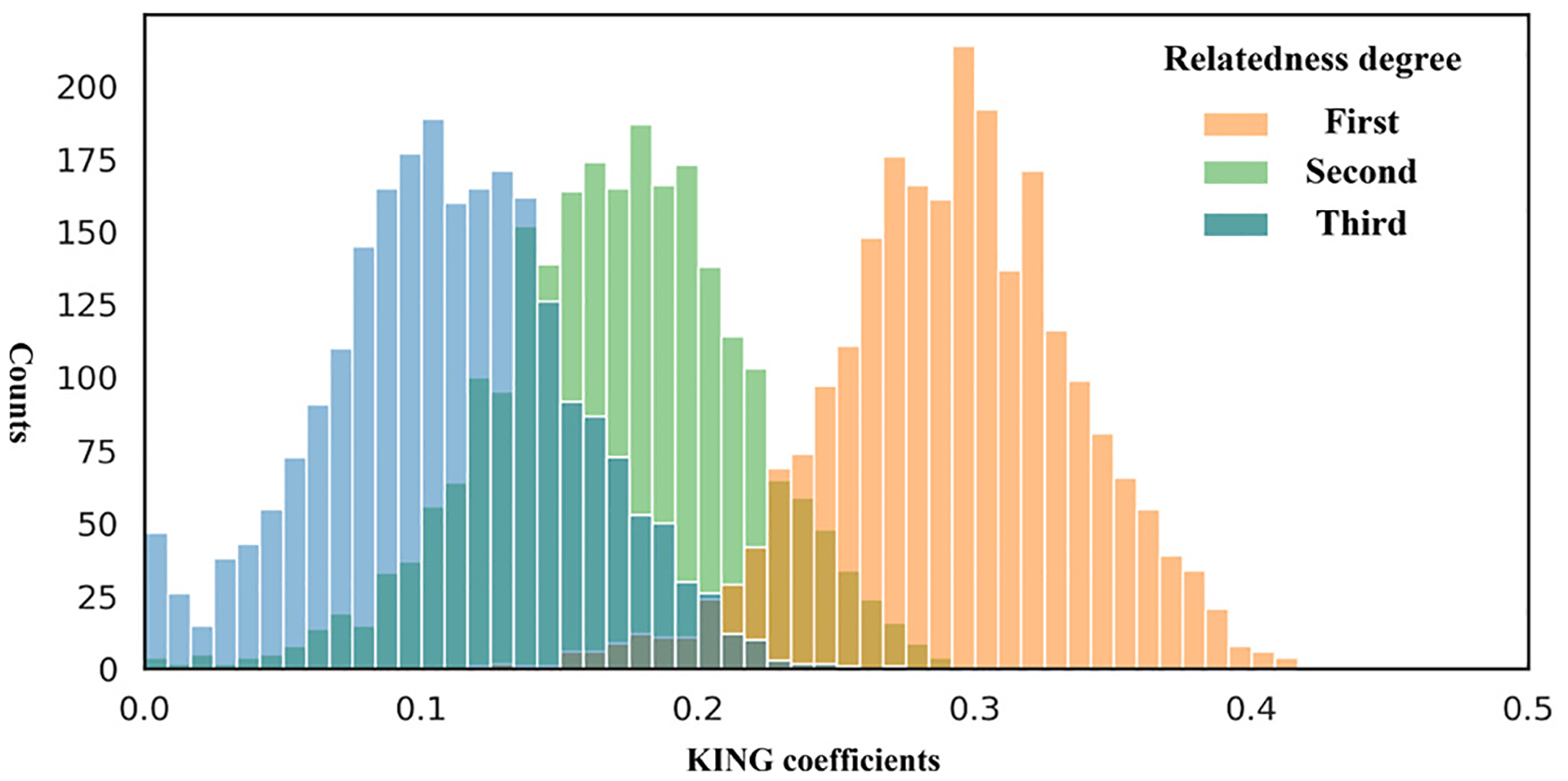
Histogram of KING coefficients between simulated relatives.

The *SynGen6* dataset provides a diverse genomic dataset, containing kinship and phenotype information across six distinct ancestry groups. Table I summarizes the key characteristics of this dataset. To validate the quality of the generated genetic markers, we compare the MAF values between the original and synthetic SNPs. As shown in Fig. 2, the distribution of MAF values for synthetic SNPs closely resembles that of the original SNPs, showing that the synthetic data preserve key allele frequency patterns. Next, we conduct a t-SNE analysis [17] on each ancestry group to assess the alignment between synthetic and original samples. Fig. 3 illustrates that synthetic samples are well aligned with the original samples across all six populations, especially in the African, American, and European ancestry groups. However, there is noticeable divergence in the East Asian group, indicating lower representation in the synthetic samples. A more comprehensive evaluation of the quality and utility of the synthetic samples is necessary and will be addressed in future work. Furthermore, to assess the quality of the synthetic relatives, we compute the KING [18] coefficient, a widely used measures for estimating kinship between individuals, and plot the distribution of kinship-relatedness coefficients in Fig. 4. The KING kinship coefficient *ϕ*(*i, j*) between two individuals *i* and *j* is defined as

$$\phi (i,j)=\frac{2n_{11}-4(n_{02}+n_{20})-n_{*1}+n_{1*}}{4n_{1*}}$$

where *n*_11_ denotes the total number of SNPs in which both individuals *i* and *j* are heterozygous, *n*_02_ refers to the total number of SNPs where *i* is homozygous dominant and *j* is homozygous recessive, *n*_20_ denotes the number of SNPs in which individual *i* is homozygous recessive and individual *j* is homozygous dominant, and *n*_1*_ and n_*1_ denote the total SNPs where *i* and *j* are heterozygous, respectively.

Fig. 4 shows that the synthetic relatives exhibit realistic kinship relationships. Last, for the watermark SNPs, which are designed to be highly associated with the phenotype vector, we conduct a logistic based test. The average *p*-value for the 20 watermark SNPs is 9.55 × 10^−36^, with a standard deviation of 3.50 × 10^−35^, indicating that these SNPs maintain a strong association with the phenotype.

## RECORDS AND STORAGE

The *SynGen6* dataset is organized into a compressed folder containing five CSV files, each serving a specific purpose related to genomic, kinship, and phenotype data. The Python and R script used to generate the synthetic dataset is also included and publicly available (see “Source Code and Scripts” section). These files are designed to facilitate comprehensive preliminary genomic analysis. The *Sample SNP Data* file contains the primary genetic information for all individuals. The *Phenotype Condition Data* file associates individuals’ genotypes with simulated phenotype. The *Watermark SNP Data* file ensures data integrity using synthetic SNPs for validation in cloud-based analyses. Finally, the *Kinship-Relatedness Data* provides information on genetic relationships between individuals. A detailed description of each file is given as follows.

It is important to note that access to the *SynGen6* dataset must be obtained via the *All of Us* Research Hub,^2^ since this dataset cannot be publicly distributed. This study used data from the All of Us Research Program’s Controlled Tier Dataset v7 available to authorized users on the Researcher Workbench. Since data generated from All of Us cannot be made publicly available, as an alternative, we provide a toy synthetic dataset generated using the same methodology but based on the *Human 10000 Genome* dataset [19], which is publicly available and can be distributed to offer insights into the *SynGen6* dataset. The DOI provided in the article refers to this toy dataset, which can be used to understand and experiment with the data format. However, it is not recommended to use this toy dataset for benchmarking or real-world deployment, and instead the actual *SynGen6* data should be used, and this dataset can be created by running our scripts in the *All of Us* Research Hub WorkBench.^3^

### File Descriptions

#### Sample SNP Data (CSV)

This file contains the SNP data for all individuals in the dataset. Each row corresponds to a unique individual.

1. *Column 1: Sample ID*: A unique identifier for each individual.
2. *Column 2: Ancestry*: The ancestry group (e.g., African and European).
3. *Columns 3: Onward*: Each column represents a specific SNP, with values reflecting the genotype (e.g., 0, 1, and 2).

#### Phenotype Condition Data (CSV)

This file contains phenotype information for each individual.

1. *Column 1: Sample ID*: Unique identifier for each individual.
2. *Column 2: Phenotype Condition*: A binary variable representing the presence (1) or absence (0) of the simulated condition.

#### Watermark SNP Data (CSV)

This file includes the synthetic watermark SNPs designed to ensure data integrity.

1. *Columns 1–20: Watermark SNPs ID*: Synthetic SNPs used for validation purposes.
2. *Row 1–30 000: Sample ID*: Each row represents the SNPs values for each sample.
3. *Row 30 001: p-Values*: The p-values indicate the statistical association between each watermark SNP and the phenotype condition.

#### Kinship-Relatedness Data (CSV)

This file provides information on the synthetic relatives in the dataset.

1. *Column 1: Sample ID*: The ID of the synthetic individual related to a sample in the *Sample SNP Data* file.
2. *Column 2: Ancestry*: The Sample ID from the *Sample SNP Data* to which the synthetic individual is related.
3. *Column 3: Relatedness*: Presents the kinship relationship.
4. *Column 4: Kinship Coefficient*: Provides the calculated kinship coefficients between the SNP data of the synthetic individual and its ancestor.

#### Synthetic Relatives SNP Data

This file provides SNP information on the synthetic relatives in the dataset.

1. *Column 1: Sample ID*: A unique identifier for each individual.
2. *Columns 2: Onward*: Each column represents a specific SNP, with values reflecting the genotype (e.g., 0, 1, and 2).

## INSIGHTS AND NOTES

*SynGen6* is designed to enable fast and efficient preliminary analysis of genomic data while addressing critical challenges, including the lack of diversity, limited data sharing, and data scarcity. First, with known association relationships, the dataset facilitates benchmarking of case-control GWAS algorithms, providing a controlled environment for testing. Second, by incorporating samples related by kinship, the dataset supports research into kinship inference methods, which are often challenging to conduct due to the scarcity of such data. Third, with the introduction of watermark SNPs, it can be used to verify the integrity of the results in cloud-based computations. Finally, the synthetic samples are generated using real samples, with the added layer of LDP to safeguard individual privacy from the original dataset.

While *SynGen6* is designed to reflect realistic genomic data distributions, certain simplifications were intentionally made to ensure feasibility in its creation and design. The dataset is strictly intended as a research benchmark, with known ground truth relationships, and results derived from it are neither intended to generalize to real-world genomic data analysis nor for the deployment in real settings. Future work aims to develop a comprehensive evaluation framework that includes statistical comparison and privacy evaluations and to incorporate additional features, such as demographic information, phenotype vector infusion, and synthetic clinical data, potentially leveraging large language models (LLMs) to enhance its applicability.

## SOURCE CODE AND SCRIPTS

The source codes and scripts used in this work are available in the GitHub repository *idsla/SynGen6*.^4^

Data processing: Plink 1.9.^5^

Phenoyte simulation: *PheynotypeSimulator*.^6^

The authors have declared no conflicts of interest.

## Figures and Tables

**TABLE I.** Dataset Summary

	Original Data	Original Data (After QC)	Synthetic Data
#Samples	245 394	172 644	32 100^†^
#SNPs	202 959	7180	7200*
European (%)	54.4	51.3	16.6
African (%)	23.2	25.5	16.6
American (%)	18.3	18.6	16.6
East Asian (%)	2.3	2.7	16.6
South Asian (%)	1.3	1.4	16.6
West Asian (%)	0.4	0.4	16.6

Note:

† Including 2100 synthetic relatives.

* Including 20 watermark SNPs.

## Footnotes

1 Phenotype Simulation section in https://github.com/idsla/SynGen6/blob/main/gen_allofus.ipynb

2
https://workbench.researchallofus.org/

3 Please see https://www.researchallofus.org/frequently-asked-questions/#workbench-faqs for information regarding how to access All of Us WorkBench.

4
https://github.com/idsla/SynGen6

5
http://pngu.mgh.harvard.edu/purcell/plink/

6
https://cran.r-project.org/web/packages/PhenotypeSimulator
